# Model-based spatial navigation in the hippocampus-ventral striatum circuit: A computational analysis

**DOI:** 10.1371/journal.pcbi.1006316

**Published:** 2018-09-17

**Authors:** Ivilin Peev Stoianov, Cyriel M. A. Pennartz, Carien S. Lansink, Giovani Pezzulo

**Affiliations:** 1 Institute of Cognitive Sciences and Technologies, National Research Council, Rome, Italy; 2 University of Amsterdam, Swammerdam Institute for Life Sciences–Center for Neuroscience Amsterdam, The Netherlands; Brain and Spine Institute (ICM), FRANCE

## Abstract

While the neurobiology of simple and habitual choices is relatively well known, our current understanding of goal-directed choices and planning in the brain is still limited. Theoretical work suggests that goal-directed computations can be productively associated to model-based (reinforcement learning) computations, yet a detailed mapping between computational processes and neuronal circuits remains to be fully established. Here we report a computational analysis that aligns Bayesian nonparametrics and model-based reinforcement learning (MB-RL) to the functioning of the hippocampus (HC) and the ventral striatum (vStr)–a neuronal circuit that increasingly recognized to be an appropriate model system to understand goal-directed (spatial) decisions and planning mechanisms in the brain. We test the MB-RL agent in a contextual conditioning task that depends on intact hippocampus and ventral striatal (shell) function and show that it solves the task while showing key behavioral and neuronal signatures of the HC—vStr circuit. Our simulations also explore the benefits of biological forms of look-ahead prediction (forward sweeps) during both learning and control. This article thus contributes to fill the gap between our current understanding of computational algorithms and biological realizations of (model-based) reinforcement learning.

## Author summary

Computational reinforcement learning theories have contributed to advance our understanding of how the brain implements decisions—and especially simple and habitual choices. However, our current understanding of the neural and computational principles of complex and flexible (goal-directed) choices is comparatively less advanced. Here we design and test a novel (model-based) reinforcement learning model, and align its learning and control mechanisms to the functioning of the neural circuit formed by the hippocampus and the ventral striatum in rodents—which is key to goal-directed spatial cognition. In a series of simulations, we show that our model-based reinforcement learning agent replicates multi-level constraints (behavioral, neural, systems) emerged from rodent cue- and context- conditioning studies, thus contributing to establish a map between computational and neuronal mechanisms of goal-directed spatial cognition.

## Introduction

The neurobiology of goal-directed decisions and planning in the brain is still incompletely known. From a theoretical perspective, goal-directed systems have been often associated model-based reinforcement learning (MB-RL) computations [1,2]; yet, a detailed mapping between specific components (or computations) of MB-RL controllers and their brain equivalents remains to be established. Much work has focused on brain implementations of single aspects of MB-RL controllers, such as action-outcome predictions or model-based prediction errors [3,4]. A more challenging task consists in mapping MB-RL computations to a systems-level neuronal circuit that provides a complete solution to decision and control problems in dynamic environments–or in other words, identifying the biological implementation of a complete MB-RL agent rather than only one or more components.

**Fig 1:**
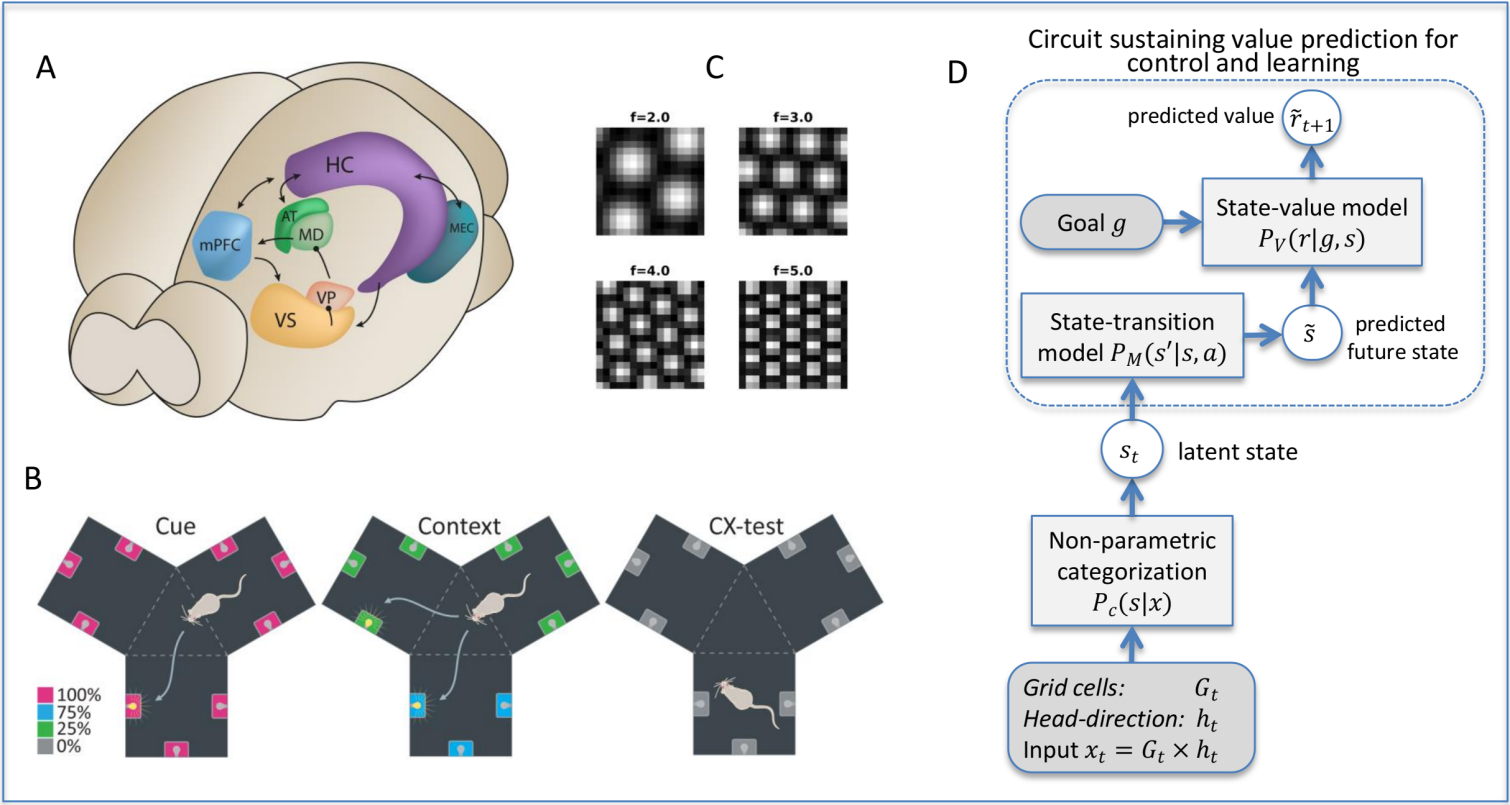
Spatial navigation in rodents: functional organization, scenario, and overall architecture of the model. (A) Structures in the rodent brain that are involved in goal-directed navigation. HC-VS constitute the essential structures of the putative model-based control system, which supports goal-directed behavior. AT and MEC provide input to the model-based control system. Output of the HC-VS circuitry may reach the cortex/mPFC via VP and MD. (B) The Y-maze used here and in [19]. Each room contains 3 goal locations, which can be cued with a light with different probability in three different phases (separate panels) according to the color legend. See the main text for more explanation. (C) Sample grid cells providing systematic (spatial) information about the environment. (D) Architecture of our biologically inspired model-based reinforcement-learning model for spatial navigation that combines non-parametric clustering of the input signal P_c_(s|x), a state-transition model P_M_(s′|s,a), and a state-value model P_V_(r|g,s) with lookahead prediction mechanism for learning and control. See the Methods for details. Abbreviations: AT, anterior thalamus; HC, hippocampus; MD, mediodorsal thalamus; MEC, medial entorhinal cortex; mPFC, medial prefrontal cortex, VP, ventral pallidum, VS, ventral striatum.

The neuronal circuit formed by the (rodent) hippocampus and ventral striatum circuit is particularly appealing, and can be productively taken as a “model system” to understand biological implementations of model-based computations during spatial navigation (see Fig 1A). The hippocampus (HC) has long been implied in place-based and goal-directed navigation [5]. Recent findings suggest that the role of hippocampus in goal-directed navigation may be mediated by the strong projections from the hippocampal CA1 and subicular areas to the ventral striatum (vStr) [6], which might convey spatial-contextual information and permit forming place-reward associations [7–10]. From a computational perspective, the hippocampus and ventral striatum may jointly implement a *model-based controller* for goal-directed choice [8,11–17]. In this scheme, HC and vStr might be mapped to the two essential components of a model-based reinforcement learning (MB-RL) controller [1,18]: the *state-transition model*, which is essentially a model of the task that permits to predict the next location given the current state (say, a given place) and chosen action, and the *state-value model*, which encodes the (expected) reward associated to each state, respectively.

Different from a model-free controller, a model-based controller can use an explicit form of look-ahead prediction (or internal simulation) that permits to imagine future trajectories and covertly evaluate them [1]. Similar look-ahead predictions have been reported in the HC: at difficult decision points, such as when they are at the junction of a T-maze, rodents sometimes stop and produce *internally generated sequences* of neuronal activity in the HC that resemble the sequential neuronal activity observed in the same area when they navigate through the left or right branches of the T-maze [7,20]. These internally generated sequences may serve to serially simulate future spatial trajectories (e.g., a trajectory to the left and successively a trajectory to the right). In turn, these look-ahead predictions might elicit covert reward expectations in the vStr [9]. By linking spatial locations with reward information, the HC-vStr might thus jointly implement a model-based mechanism that allows an animal to covertly simulate and evaluate spatial trajectories [13,21], using a serial scheme that has some analogies with machine learning algorithms (e.g., forward simulations in Bayes nets [22,23] or Monte Carlo rollouts in decision trees [24]). Internally generated sequences were also reported during sleep or rest periods in the hippocampus and associated structures such as the ventral striatum [25]; in this case, they have been associated with multiple functions such as planning [26] and the off-line “replay” of past trajectories for memory consolidation [27,28]–a mechanism that has inspired early (DYNA [29]) as well as more recent (“experience replay” [30]) machine learning schemes.

Here we present a biologically-grounded computational analysis of model-based RL, by comparing side-by-side the behavior and neuronal activity of living organisms (rodents) and of a probabilistic MB-RL agent, in *cue*- and *context- conditioning* tasks that depend on intact hippocampal and ventral striatal (shell) function [19,31,32]. The results we report indicate that the MB-RL agent replicates the multi-level constraints (behavioral, neural, systems) emerged from rodent cue- and context- conditioning studies. First, we show that the MB-RL agent correctly learns to obtain reward in multiple goal locations and develops contextual preferences that are analogous to those of rodents. Second, we show that latent states emerging in the *state-transition* and *state-value* models of the MB-RL agent show key coding properties of HC and vStr neurons, respectively. Third, by comparing multiple variants of the MB-RL algorithm, we show that forward (look-ahead) predictions—of the kind that can be associated to hippocampal forward sweeps [20]—improve both action selection and learning. This latter result is important as the computational efficacy of model-based schemes based on forward sweeps has been put into question [33].

Taken together, the results of this study speak to the possibility of aligning the MB-RL scheme to the HC—vStr circuit to reproduce both behavioral and neuronal data. Establishing this kind of mappings is particularly important to foster cross-fertilizations between reinforcement learning and neurophysiology; for example, to identify specific algorithmic solutions that the brain uses to face problems that are still challenging in machine learning, or conversely, to advance novel computationally-guided theories of neural processing [10,34].

Finally, our MB-RL scheme has two main features that go beyond the state of the art from a computational perspective, and which are necessary for an adaptive agent to deal with open-ended situations. First, the MB-RL agent successfully faces the challenge to learn simultaneously three components: (the latent categories that form) the state space, the state-transition and the state-value models. Our novel scheme that combines non-parametric and reinforcement learning ensures that only the latent categories that afford accurate state-transition and state-value functions are retained, thus linking inextricably the three learning processes. Second, the MB-RL agent can deal with multiple goals in a native way, without the necessity of re-learning.

## Results

We report the results of a series of simulations, which aim to assess whether the novel MB-RL agent introduced here replicates the multi-level constraints (behavioral, neural, systems) that emerge from a context conditioning task, in which neural ensembles in rat hippocampus and ventral striatum were simultaneously recorded [19].

### Experimental set-up: Y-maze

The maze used in the animal study and in our simulations is a y-shaped symmetric arena consisting of 3 identical square chambers rotated 120 degrees from each other and connected through a central triangular passage, see Fig 1B. Each chamber contained three goal locations located along the chamber walls, where reward was (probabilistically) delivered. Each reward location had a cue light above it.

Our simulation follows the protocol used in the animal study [19] and consists of three phases. In the first phase (*Cue*: *cue conditioning*), the correct goal location was cued with a light; reward was delivered if the animal reached the goal location and made a nose-poke (but note that we do not simulate the nose poke here). In the second phase (*Context*: *contextual conditioning*), the correct goal location was cued with a light, too; but the probability of reward (following a nose poke) depended on the chamber’s spatial position: south room goals brought reward in 75% of the trials, whereas the other goals brought 25% reward. Finally, a *context-conditioning test* (*CX test*) was performed, with no cues or rewards (i.e., "free run"), to probe the animals’ acquired contextual place preferences.

The key behavioral result of the animal study, which we aim to reproduce in the MB-RL agent model, is that the contextual preference for the south room goals is preserved in the last (CX test) phase. We also aim to test whether the internal representations acquired by the two key components (*state-transition* and *state-value* model) of the MB-RL agent during learning, have coding properties that resemble HC and vStr activations, respectively, in the animal study.

### Brief introduction to the computational model

The computational model (MB-RL) is fully explained in the Methods section; however, we shortly summarize it here for the sake of the reader. The model essentially includes three interconnected components: a latent state categorization mechanism, which permits to learn the state representations that are useful to solve a task (e.g., place cells); a state-transition model, which learns the contingencies between actions and future states (e.g., what location do I reach by going left?) and a state-value model, which learns the utility of the states (e.g., is reaching location x good or bad?).

There are three important aspects of the model to note (see the Methods section for further details). First, we assume that state-transition and state-value models correspond to HC and vStr, respectively, and they jointly permit to steer look-ahead predictions (analogous to hippocampal forward sweeps [20]). As we will discuss below, our simulations will compare different versions of the same MB-RL agent, which use, or not use, look-ahead predictions for learning, control (action selection) or both.

Second, the MB-RL agent includes a mechanism that adaptively selects whether or not to do a sweep, and the depth of the sweep, depending on the agent's uncertainty and confidence about the choice. Essentially, the depth of sweeps decreases when the agent becomes sufficiently confident about its choice. However, a change in reward contingencies (e.g., the location of reward) would produce a reversal of this effect—and the generation of longer sweeps. This is because after failing to reach a reward at the expected location, the agent would become again uncertain about its choice, hence triggering sweeps to minimize it before a choice [23]. Here we are interested in validating the computational efficiency of this mechanism, which may potentially explain why VTE behavior increases rapidly when animals make errors following a switch in reward contingency [20].

Third, most model-based planning algorithms start from a state space that is predefined by the programmers. However, the brain has to simultaneously learn internal models to generate predictions, and the states to be used by the model. In keeping, in the MB-RL agent, the learning processes required to acquire latent states and the (state transition and state value) models that use the latent states are interdependent (see Eq 1 below). Here we are interested in validating this learning scheme, which permits learning latent states that not only entail high perceptual accuracy but also afford good prediction and control when used by the state-transition and state-value models.

### Behavioral analysis: Accuracy

Successful context conditioning was evaluated in [19] with a *CX test*: a “free-run” target preference behavioral test in which the rats freely explored the maze for several minutes without any cue or reward. During this period, to probe the animals' learned preferences, statistics were collected about their visitations of (and “nose pokes” in) rooms that in the previous *Contextual Conditioning* phase were associated with high- and low-reward probabilities. Analogously, to test the MB-RL agent’s learned preferences, we ran a free-run simulated test for 2,000 time steps, in which the agent started from a small central area (3x3 units) and with random orientation. In this simulation, action selection maximized the likelihood for (expected) reward pooled from all the 9 goal locations; and the agent did not learn. To better clarify the behavioral effects of conditioning in the MB-RL agent, we performed the same free-run test (and without learning) also at the end of cue conditioning. Finally, and importantly, we envisaged to compare different versions of the same MB-RL agent that uses, or not uses, look-ahead predictive mechanisms (analogous to hippocampal forward sweeps [20]) for both control / action selection and (reward) learning—in order to assess the computational importance of these mechanisms. Specifically, we compared four different versions of the MB-RL agent using a 2x2 design, which considers 2 controllers (i.e., using or not using forward sweeps) and 2 learning procedures (using or not using forward sweeps); see Section 4 for details.

**Fig 2:**
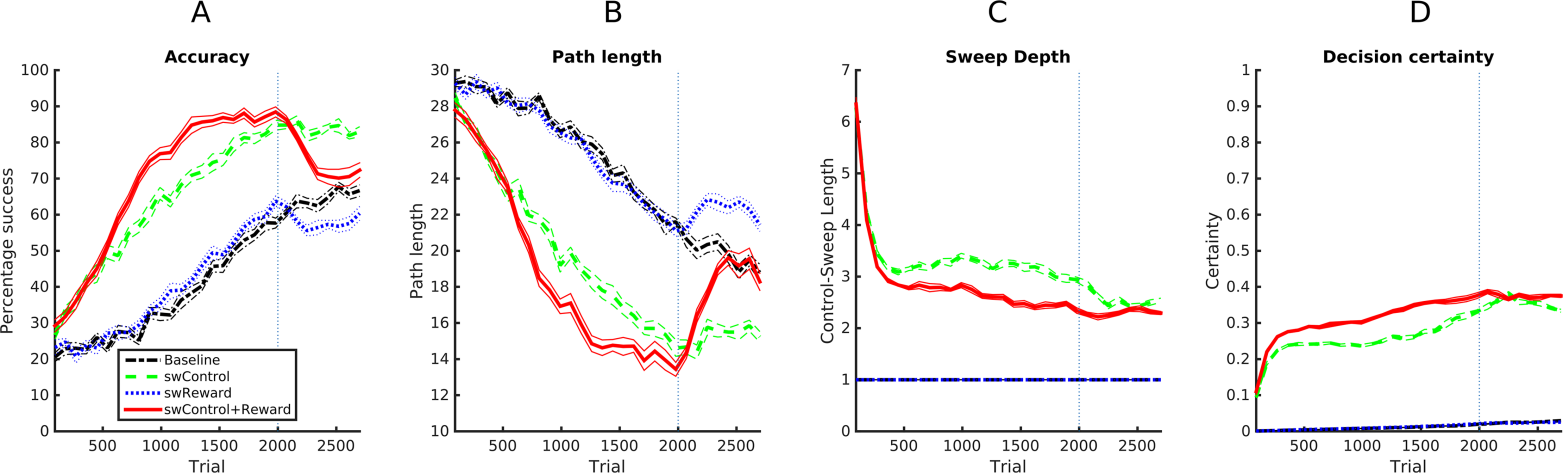
Benefits of forward sweeps for action selection and control. (A,B) Learning performance (accuracy and length of the agent path to the goal). (C) Length of sweeps used for control / action selection. (D) Decision (un)certainty during control / action selection.

We expected that look-ahead prediction (or forward sweeps) to provide the agent significant advantages for both control / action selection and (reward) learning; this result would be particularly intriguing for action selection, given that the baseline controller uses the full factorized distribution of reward and is thus computationally demanding. The results shown in Fig 2 confirm our hypotheses, by showing an additive advantage of forward sweeps in both action selection and learning. As shown in Fig 2A, the MB-RL agent that uses forward sweeps in both action selection and learning (swControl+Reward), with average success of μ = 69% (σ = 2) during the entire *Cue Conditioning* phase outperforms in amount of collected reward the agent that uses forward sweeps only for control (swControl, μ = 62% (σ = 3), t(1,9) = 6.9, n = 10, p<0.001), the agent that uses forward sweeps only for learning (swReward, μ = 39% (σ = 2), t(1,9) = 33.4, n = 10, p<0.001), and the agent that lacks them during both control and learning (Baseline, μ = 36% (σ = 1), t(1,9) = 39.6, n = 10, p<0.001) and this advantage is evident from the very beginning of learning. For all the four agents, performance decreases after the vertical dotted bar, in correspondence of the *Contextual Conditioning* phase—but this is expected, given that less reward is available in this phase.

Fig 2B illustrates the same advantage in overall path length needed to reach the reward site of the four agents. Along with the increased performance, the length of sweeps used for control by the swControl+Reward and swControl agents decreases with time (Fig 2C). This is because the agent’s decision certainty progressively increases (Fig 2D) as their state-value model becomes more effective; in turn, the mechanism for sweep length control explained in the Methods Section decreases length (we obtained equivalent accuracy and path length results using a controller having a constant length of 9 and thus requiring more computational results; results not shown). This result aligns well with evidence that hippocampal forward sweeps at decision points progressively diminish during learning [20]—until they disappear when the animal develops a habit (which we do not model here, but see [13]).

### Behavioral analysis: Preferences after conditioning

As the above analysis confirmed the advantages of the MB-RL agent that uses forward sweeps in both action selection and learning (swControl+Reward), we used this MB-RL agent for the rest of our behavioral and neural analyses.

**Fig 3:**
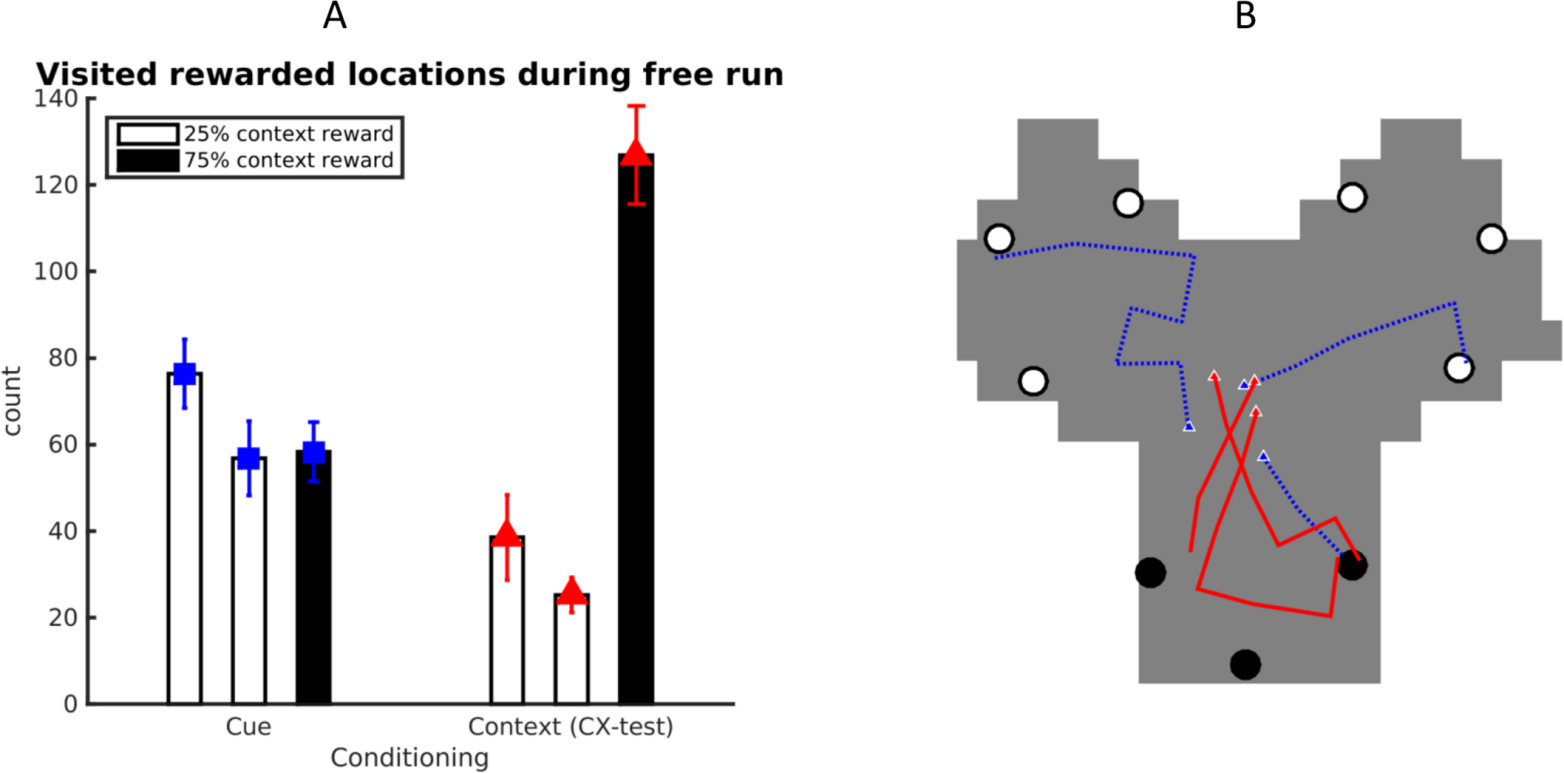
Behavioral results of the simulations. (A) Preferences of the MB-RL agent after *Cue Conditioning* and after Contextual conditioning (i.e., *CX test*). In the CX test, both agent and animals show a preference to visit targets in the room that was previously most rewarded. (B) Sample trajectories of the agents during these tests (after *Cue Conditioning*, blue; after Context Conditioning, or *CX test*, red).

Our target rodent study reported that during "free run" in the *CX test* phase, animals showed an increased preference for the room that yielded more rewards in the previous (*Context*) phase [19]. The MB-RL agent correctly reproduces this pattern of behavioral results. As shown on Fig 3A, in the *CX test* phase (in the absence of reward), the agent shows a significant behavioral preference (number of reward-site visits) for the room that was previously most rewarded (room1 vs. room3, t(1,9) = 4.3, n = 10, p = 0.002; room2 vs. room3, t(1,9) = 7.6, n = 10, p<0.001), which matches the animal's learned preference (and differed from an analogue test executed right after cue conditioning: room1 vs. room3, t(1,9) = 1.5, n = 10, p = 0.16; room2 vs.–room3, t(1,9) = 0.1, n = 10, p = 0.92). Fig 3B provides three representative trajectories before (blue trajectories) and after (red trajectories) context conditioning–the latter exemplifying the increased preference for the room that included highly rewarded locations in the Context phase (black dots).

### Neural-level analysis of conditioning

To further establish a parallel between the MB-RL agent and the HC—vStr circuit, we analyzed the *content* of the latent states that emerged in the agent’s *transition model P*_*M*_(*s*′|*s*,*a*) and *value function P*_*V*_(*r*|*g*,*s*) at the end of the *Cue* and *Context Conditioning* phases. We decoded the spatial location corresponding to each latent state and created: (a) value maps for each target showing for each spatial location the greatest reward across all head-directions and (b) transition maps showing how position could change after applying an(y) action at any orientation. We also analyzed the effect of conditioning at the neural level: how the learned internal models change and how this change affects behavior.

**Fig 4:**
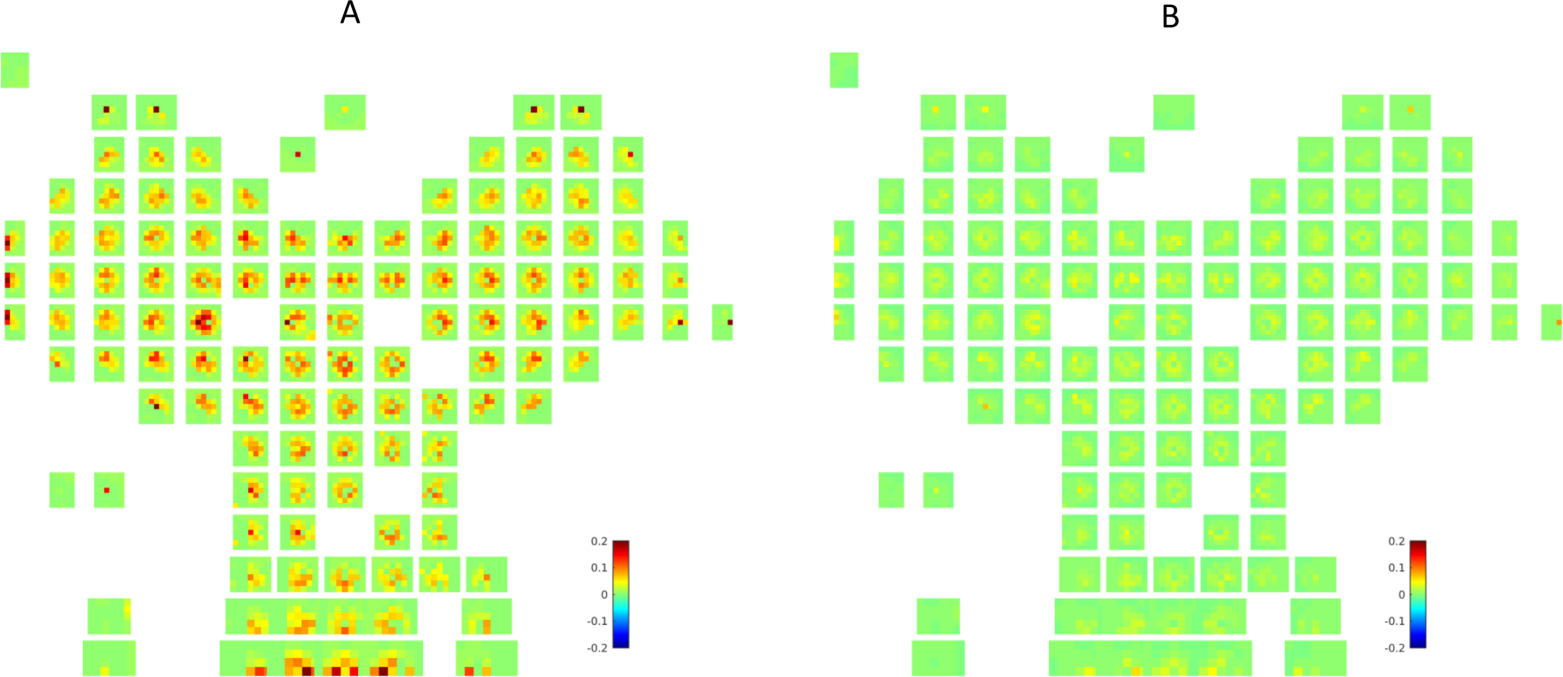
Neural representation of state transitions in the state-state model. Latent states developed by the state-transition models averaged across all 10 learners after the *Cue Conditioning* phase (A); and the changes due to *Contextual Conditioning*, i.e., the differences between probabilities before and after *Contextual Conditioning* (B). Each image from the transition model *P*_*M*_(*s*′|*s*,*a*) encodes the greatest likelihoods *P*_*M*_(*xy*(*s*′)|*xy*(*s*), *a*) across all head-directions and actions to step from the location *xy*(*s*) of a given latent state (place) *s* to nearby places *xy*(*s*′) located within the range of an(y) action from the location of the current place, following any of the available actions, i.e., the (probabilistic) location of the successors of every state. The locations *xy*(*s*) and *xy*(*s′*) of *s* and *s*′ are decoded using an inverse of the function providing input to the Dirichlet model. Note that, as expected, the decoding procedure is not perfect—hence the gaps in the maps.

The neuronal analysis of HC and vStr cells after the cue conditioning task reported different coding characteristics in the two areas, with the former showing high spatial selectivity and the latter showing selectivity for reward- and task-related information, and place/reward combinations [19]. The two components of the MB-RL agent acquire analogous coding preferences after conditioning. Fig 4 shows the transition probabilities learned by the state-state (transition) model after *Cue Conditioning* (Fig 4A) and how they changed after *Context Conditioning* (Fig 4B). In Fig 4, each green square corresponds to a place in the maze (i.e., the central square corresponds to the central place of the maze) and the colors of the smaller squares code the greatest probability of transitioning from the location of that state, regardless the direction, to the location of any other state of the maze (shown are only the possible successors, i.e., nearby locations), by executing any of the 3 actions available to the agent (see Methods section). The more red the color of the cell, the higher the probability. In other words, this map shows the spatial projection of the learned "successor states" (small squares) of each latent state (green squares) implicitly coded in the agent's probabilistic transition model—analogous to "successor representations" [35,36]. For example, the green squares near the center include transitions to all directions while the green squares on the borders only include transitions towards the center. In other words, the learned transition probabilities encode the actual transitions available in the environment, reflecting the idea of HC encoding a navigational "cognitive map" [5]. Furthermore, importantly for our analysis, these codes only change (remap) to a minor extent between the *Cue* and *Context Conditioning* phases (Fig 4B)—in keeping to what was reported empirically [19]. Essentially, the changes reflect a consolidation of the same transition model, not a remapping, due to additional training during the *Context conditioning* phase.

**Fig 5:**
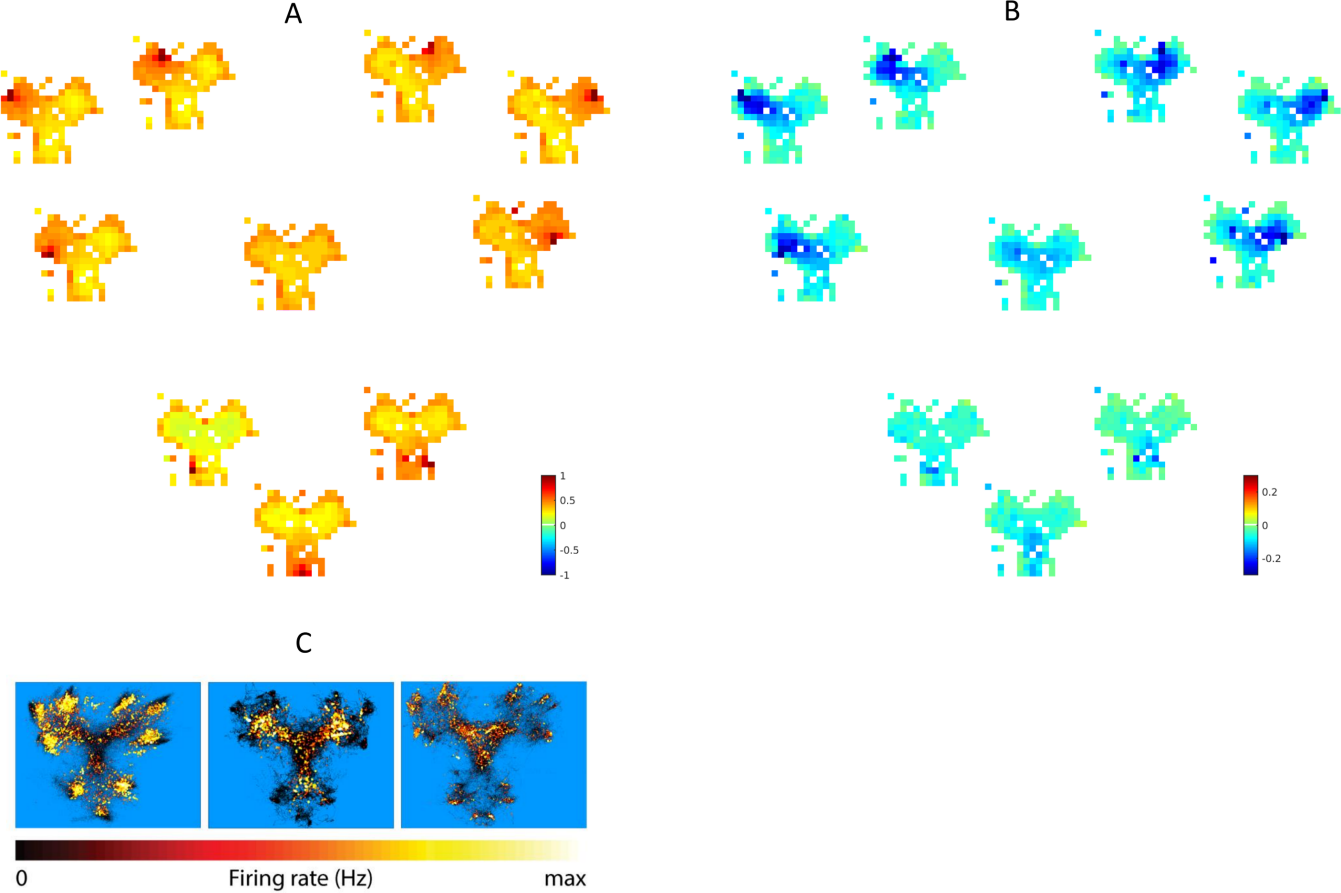
Neural representation of value in the state-value model. Latent states developed by the state-value model averaged across all 10 learners, after the *Cue Conditioning* phase (A); and how they change after *Contextual Conditioning*, i.e., the differences between probabilities before and after *Contextual Conditioning* (B); and the activity of three sample vStr neurons drawn from the experiment in [19] (C). The images in (A, B) represent the greatest value *P*_*V*_(*r* = 1|*g*,*xy*(*s*)) across all head-directions attributed to a given spatial position *xy*(*s*) for a given target *g*. Each image represents one target and its location in the plot corresponds to its location in the Y-maze. The central image represents the combined value function across all targets. It is rotationally symmetric after the rotationally symmetric reward delivered during Cue Conditioning (as some of the vStr rodent-neurons; see insets C) and becomes asymmetric during the context conditioning phase. The spatial positions *xy*(*s*) are decoded from the latent states *s* using an inverse of the function providing input from the grid cells.

Fig 5 shows the value of each place (i.e., the learned probability of obtaining reward in the place) when a goal location is selected or cued, as learned by the state-value function component of MB-RL agent after *Cue Conditioning* (Fig 5A) and how these values changed after *Context Conditioning* (Fig 5B). The nine outer big Y-shapes represent the possible goal sites; inside each of them, the colors code the probabilities of obtaining reward while starting from any specific location in the maze—the darker red the color, the higher the probability. The central Y-shape represents the combined value function across all goal sites, which is effective when the goal is not cued, as in the behavioral CX test. These probabilities relate well with key coding properties of vStr neurons in [19]. If one assumes that the current goal is unknown, as in the CX-test, one can see the same rotational invariance in the model (Fig 5A, the central Y-shape) as in sample vStr neurons (Fig 5C; for more samples, see [19]). This aligns well with the idea that vStr neurons might encode place-reward associations and these in turn can be conditioned on a specific goal, possibly provided by prefrontal areas [37] or vicariously from hippocampal forward sweeps [21,38].

Note that the probabilities emerging in the state-value model are closely related to reward functions of RL, which can be used to learn a policy from the current state [1] and, in a model-based scheme, to retrieve covert expectations of reward using mental simulation or forward sweeps [13,21,23]. As usual in RL, the value of reward places is temporally discounted such that places farther from goal sites have lower values. This creates a sort of gradient or "tessellation" of the task, which might reflect not only proximal distance but also other information such as phases or subtasks that are necessary to secure a reward [19].

**Fig 6:**
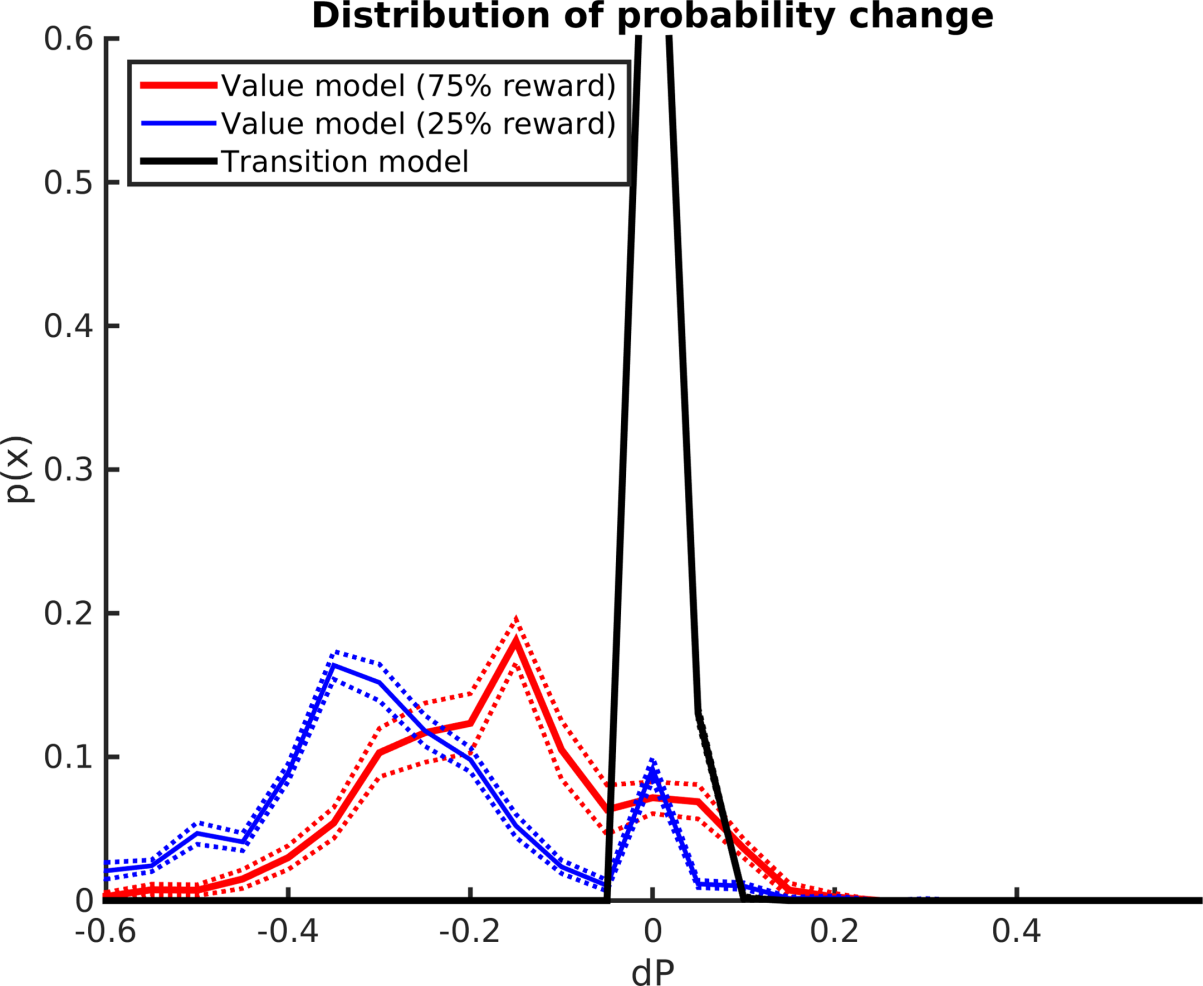
Changes in the state-state and state-value models after contextual conditioning. This figure shows what changes the Contextual Conditioning procedure produces in the probability values of states in the state-state or transition model (black), and in the state-value model, where states correspond to the most rewarded (red) or less rewarded (blue) rooms. For clarity, we only show the changes of states having a probability that is greater than 0.05 (for the state-state model) and 0.5 (for the state-value function). This choice of thresholds in motivated by the fact that in the state-value function we are interested in verifying changes in the states carrying significant value information (e.g., those regarding the goal states or their neighbor’s), not in the many states that have a low probability value in all situations (see Fig 5).

Importantly, the coding of value in the state-value model changes drastically after *Contextual Conditioning* (Fig 5B). While after Cue Conditioning one can observe full rotational symmetry, after the Context Conditioning a marked preference is evident for the goal locations in the most rewarded (lower; south) room—which also explains the behavioral results of Fig 2. This change of preference can be better appreciated if one considers the distribution of the change of values associated with latent states in the state-value model after *Contextual Conditioning* (Fig 6). Specifically, there is a decrease of all the state values (given that reward is less frequently available), but the value of states corresponding to the less rewarded rooms (blue, mean change of -0.29 across all learners) decreases significantly more than those of the more rewarded rooms (red, mean change of -0.17 across all learners; t(1,9) = 25.0, p<0.001). This result is coherent with a body of literature implying vStr in the coding of reward expectancies [21] and would imply a sensitivity for changing reward contingencies (see Discussion). This is in sharp contrast with the states in the state-state transition model (black), which essentially does not change (mean change of 0.01 across all learners).

**Fig 7:**
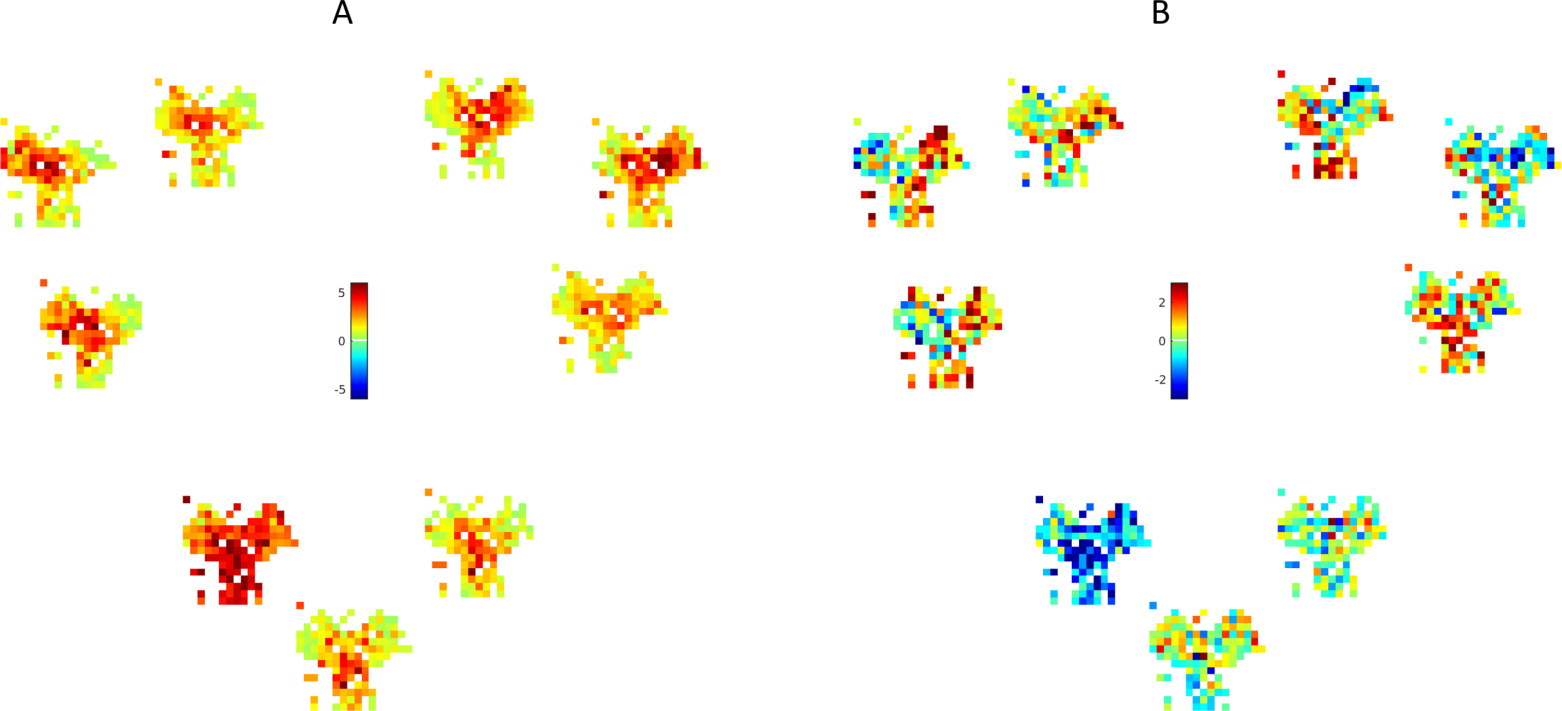
Analysis of sweeps. Length of sweeps during control / action selection, for each target (separate Y-maze images) and each spatial location (dots in the Y-mazes). Sweep length is color-coded. (A) Sweep length after *Cue Conditioning*. (B). Change of sweep length after *Context Conditioning*.

Finally, Fig 7 provides analysis of the sweeps for action selection generated during the two conditioning phases. During *Cue Conditioning*, sweep length increases at decision points, e.g., the center of the maze and nearby densely located targets (Fig 7A). This simulates the rodent data that consistently show longer internally generated sequences at branch points [15], [23]. In turn, Fig 7B shows how sweep length changes after *Context Conditioning*. Notably, when the agent is in a location that is far away from targets with low reward probability, it needs longer sweeps to accumulate sufficient evidence about the most valuable action. Instead, sweep length remains the same or decreases for highly rewarded targets.

## Discussion

Computational methods of reinforcement learning have been widely used to analyze neuronal data [2,39–41]. However, this has been done far more systematically for model-free RL–mostly associated to habitual behavior—than for model-based RL methods [22,23,42]–more associated to goal-directed behavior and planning. A challenge consists in mapping the components of a MB-RL agent to a neural circuit that offers a complete, systems-level solution to goal-directed choice, rather than a single aspect of it (e.g., the dynamics of dopaminergic neurons and their relations to reward prediction error).

Rodent research suggests that the neuronal circuit formed by the hippocampus (HC) and ventral striatum (vStr) might implement model-based computations in spatial navigation. In this scheme, the HC learns a state-transition model (permitting to predict future states) and the vStr learns a state-value model (encoding goal-dependent state values). The HC might then predict future trajectories (e.g., using forward sweeps and ripple-associated replay) and vStr might covertly evaluate them, permitting to select the best (that is, reward-maximizing) course of action [12,13,21]. To provide a computationally-guided analysis of this idea, here we designed a Bayesian model-based reinforcement learning (MB-RL) agent and tested it in a challenging contextual conditioning experiment that is dependent on hippocampal and ventral striatal function [19].

The results of our simulation show that the MB-RL agent procedure can reproduce animal data at multiple—behavioral, neural, systems—levels. At the behavioral level, the agent shows place preferences that are analogous to rodents [19]. Key to this result is the fact that the agent's value function is conditioned on goal information. Here, in other words, goal information acts as a “context” and permits the agent to learn multiple goals, as required by the experimental set-up [19]. This possibility is precluded to other RL controllers that are only optimized for a single value function (or goal) and thus lack context sensitivity [1] but is important to scale up RL methods to real-world situations that involve multiple potential goals. At the neural and systems levels, the agent develops internal codes that reproduce key signature of hippocampal and ventral striatal neurons [19]. This is important to establish deep relations between formal methods and neurophysiology that potentially span multiple levels of analysis—from computational to algorithmic and implementational [43]. Finally, in our proposed model, hippocampal sweeps can contribute to both action selection and learning. Our comparison of multiple MB-RL schemes shows that mechanisms of look-ahead prediction that resemble hippocampal forward sweeps improve both control and learning. There has been some recent skepticism around the idea of using look-ahead prediction for control and learning, based on the fact that prediction errors tend to accumulate over time [33], which would cast into doubt the idea that hippocampal sequences may serve predictive or planning roles. Furthermore, alternative proposals have cast model-based control (and hippocampal function) in terms of backward [42] or a combination of forward and backward inference [22]. Our results suggest that the limited form of look-ahead prediction that we adopt is computationally advantageous, thus lending computational-level support for theories that assign forward sweeps a predictive role [44,45].

The MB-RL agent is related to other computational models of model-based spatial decisions [22,23,46–52] that use various forms of forward planning. Different from these architectures, the MB-RL agent includes a nonparametric module that learns a “state space” in an unsupervised manner while the agent learns the task, and which works synergistically with the other components of the architecture. Indeed, the state space representations emerging from the nonparametric learning procedure ensure that only states that afford good prediction and control are retained. Using end-to-end learning (i.e., from perception to action) helps keeping the various learning procedures (state space, action-state and state-value learning) coordinated and is more effective and biologically realistic than using a staged approach—still popular in RL—in which unsupervised state space learning is seen as a generic preprocessing phase. From a computational perspective, this approach can be considered to be a novel, model-based extension of a family of Bayesian methods that have been successfully applied to decision-making problems [53–57]. It is also important to note that our approach does not require learning a separate state space (or sensory mapping) for each goal; rather, multiple spatial goals share the same state space—which implies that our MB-RL agent can deal natively with multiple goals. This is different from most current deep RL approaches, which require learning a new state space for each problem or task (but see [1]). In our set-up, sharing the state space is effective because all goals correspond to spatial locations—or in other words, they all lie in the same low-dimensional state space that supports spatial navigation. This reliance on a relatively low-dimensional state space makes spatial navigation quite special and tractable, both in machine learning and the brain; it is thus possible that brain areas that support goal-directed processing outside navigation domains rely on more complex computational solutions that remain to be fully established [10,58,59].

At the neurophysiological level, the latent states that emerged in the agent's state-state and state-value models during conditioning share important similarities with HC and vStr coding, respectively—and in particular the selectivity for space vs. reward-predictive information and place/reward combinations, respectively. Our computational model thus provides a detailed (yet to some extent abstract) mapping between specific components of model-based controllers and neuronal circuits. Establishing this sort of mappings is important if one aims to the cross-fertilization of current research in reinforcement learning / artificial intelligence and neurophysiology; for example, to probe in detail neuronal solutions to challenging machine learning problems. Indeed, one should expect that the neuronal networks in the HC and vStr (as well as other brain structures) have been optimized over time to solve the challenging problems that animals must face every day, and cracking this neural code might be helpful to design artificial agents with similar abilities. In this respect, a limitation of the model proposed here concerns the simplifying assumptions on HC and vStr coding and their respective roles in model-based control. For simplicity, we mapped one-to-one HC to state-transition and vStr to state-value models. However, this sharp division is likely to be simplistic and both neural coding and computational roles of the HC-vStr circuit are certainly more complex. HC cells show some sensitivity to goal and reward information [60] and remap from cue-on to cue-off and spontaneous goal site approach [19]. Furthermore, vStr can code information that goes beyond scalar reward expectations; it can code for example item- or object-specific information, such as the expected reward associated to a specific food or other characteristics of a task, including (for example) intermediate steps to goal locations [19]. This suggests a more complex architecture in which HC and vStr do not map one-to-one to components of a model-based controller but may realize model-based computations in a more distributed manner; and in which they form a latent task model that encodes task variables that are more abstract than spatial codes, permitting “tessellating” tasks into behaviorally meaningful events (e.g., task phases that lend to reward delivery) and forming hierarchies. Understanding how the brain tessellates and organizes the state space hierarchically may be particularly important for the success of future model-based reinforcement learning algorithms. Current model-based RL methods suffer from the problem that, during long look-ahead searches, errors sum up; for this, in practice, most practical applications use model-free solutions. However, in principle, endowing model-based methods with the ability to exploit relevant task structure (e.g., milestones or subgoals) may mitigate this problem, by making forward search more abstract and hierarchical (or “saltatory”) [61–63] or by avoiding chaining too many consecutive predictions [33]. To this aim, unsupervised state representation methods (including deep learning methods) that are able to identify latent task structure may be productively incorporated into model-based control systems—yet it remains to be studied in future research how to handle or bound (hierarchical) state representations that may solve realistic problems [36,64–68]. An alternative possibility consists in learning a more complex state space (successor representations) that afford implicit prediction [35,69]. Finally, it is important to remark that overall brain architecture for model-based spatial navigation plausibly includes other areas in addition to HC and vStr. For example, in rodents prelimbic cortex may be particularly important to decide whether or not to engage the model-based system (or to initiate a deliberative event [20,23]) and to code for spatial goals [37]. More broadly, the hippocampus cross-talks extensively with cortical areas, especially during sleep—and this cross talk can be essential to train not only the cortex as usually assumed [70] but also the hippocampus [14,15,71]. Realizing a complete, systems-level architecture for goal-directed spatial cognition remains an open challenge for future research.

In sum, our results highlight the viability of model-based methods to study goal-directed spatial navigation, in the same way model-free methods have been widely adopted to study habitual action control [22,23,42]. Our comparison of alternative MB-RL schemes shows that forward sweeps are useful for both learning and control / action selection. Indeed, our results show that the MB-RL agent that includes both kinds of forward sweeps is the most accurate and the one with lowest uncertainty. This result, again, connects well to neurophysiological evidence that implicates (prospective) internally generated hippocampal sequences to both learning / memory function [27,72] and decision [20,26]. Our approach has a number of implications from both computational and neurophysiological perspectives, which we address next.

From a computational perspective, it is worth noting that the results that we present here may be obtained by a model-free RL system, augmented in various ways (e.g., by using various goals as part of the state classification system). Two points are however important, the former related to coding parsimony, and the latter related to biological plausibility. First, separating the transition function from the value function (which is typical of model-based systems) makes the realization of multiple goals more “economic” from a coding perspective. In the classical RL approach, a Q-value function encodes the expected cumulative future reward for each state and action, but for one single goal [73]. The addition of multiple goals in the Q-value function would increase learning time dramatically. In contrast, adding novel goals to our model-based RL approach requires just learning the new value associated to each state. Second, and importantly, a pure model-free RL approach would fail in navigation tasks that are typically associated with the hippocampus, such as detour tasks [74]. This is because model-free RL methods lack the required flexibility to rapidly adapt to novel contingencies (e.g., a change of the reward or goal location, or of the state transitions, as in the case of detours).

Another interesting approach to understand predictive aspects of hippocampal coding is in terms of successor representations (SR) [35,69,75,76]—or prospective codes at the single cell level. Our approach is distinct from the successor representation (SR) approach [35,69,75,76], for four main reasons. Firstly, the two approaches target two distinct (possibly complementary) forms of predictive coding in the hippocampus. Our approach addresses predictive coding at the level of sequential activation of multiple cells, e.g., theta sequences and forward sweeps at decision points [13,20]. Rather, the SR addresses predictive coding at the level of single cells; and in particular the backward expansion of place fields (in CA1), which can be considered a form of predictive representation of future states or locations [69]. Secondly, the two approaches use two distinct computational schemes (model-based versus model-free or hybrid) to engage in predictive processing. In our model-based approach, predictions about future locations (and their value) are generated by engaging the state-transition and state-value models on-line, to perform forward sequential planning and policy selection. This requires learning a one-step probability transition model *P*_*M*_(*s*′|*s*,*a*) and a probabilistic cumulative value function *P*_*V*_(*r*|*g*,*s*). Rather, the SR approach does not require engaging an internal model for forward prediction during planning (although some variants of SR learn a model and engage it off-line, to "train" SR [29,76]). This is because a SR essentially caches a series of predictions (e.g., of future occupancies) into a single state representation. More formally, the SR approach learns the future occupancy *M*_*s*_(*s*′) function, or the probability to occupy any state *s*′ following a specific policy (standard, on-policy method) or any policy (extended, off-policy method [76]) starting from state *s*. Using this function, the SR approach can be sensitive to future events without engaging a model on-line. Thirdly, our approach and the SR have distinct trade-offs. Our model-based approach has the highest flexibility (because all knowledge embedded in the model is used on line) but also the highest computational cost. The usefulness of SR rests on the possibility to decompose the cumulative value function into a product of SR and local reward. The SR approach permits using predictive representations in a model-free manner, thus skipping (costly) model-based computations during planning and choice. Extensions of the SR approach permit to have roughly the same flexibility as model-based approaches, in challenging situations like detour and revaluation tasks [76]. At the same time, a prediction generated using a SR is usually less specific than a model-based prediction, as the SR marginalizes over all possible sequences of actions. Finally, and most importantly here, the two approaches would assign different roles to internally generated hippocampal sequences. Our model-based approach uses internally generated hippocampal sequences for both learning and on-line decision-making and planning. Rather, in the SR approach internally generated hippocampal sequences are not required for on-line decision-making or learning (although some variants of SR use sequences for off-line training, i.e., experience replay [29,76]). In most navigation scenarios, decisions can be done using a single SR (comparable to a single place cell or a small population of place cells [69]), hence this approach would not explain per se why the hippocampus should encode theta sequences, after sufficient learning. Of course, the two forms of prediction entailed by our model-based and the SR approach are not mutually exclusive, but can be productively combined; for example, by using sequences of SR (rather than "standard" place cells) within a model-based scheme. The efficiency and biological plausibility of such combined scheme remains to be tested.

At the neurophysiological level, the proposal advanced here is that the hippocampus encodes a model or cognitive map of spatial transitions and uses this model for state estimation (self localization) and forward inference (prediction / planning). In our scheme, these computations are intimately related. The same model can support different functions (e.g., self localization, forward or even retrospective inference), depending on the specific "message passing": state estimation may rely on the cross-talk between input structures of the hippocampus that encode the observations or inputs of the model (putatively, LEC and MEC) and the hippocampus proper (putatively, dentate gyrus and CA3-CA1) [22]; forward prediction is more dependent on the hippocampus proper (recurrent dynamics in CA3, and CA3- CA1 connections) [13,14,22]; whereas a complete goal-directed decision requires the interplay of the hippocampus with other brain areas that may putatively encode goal states (mPFC [77,78]) and/or state values (vStr [19,25]). The complete systems-level circuit supporting goal-directed decisions has not been systematically mapped. However, various studies point to the importance of dorsolateral and ventromedial prefrontal cortex, and orbitofrontal cortex in supporting model-based computations [3,79] (but see [80] for evidence that orbitofrontal cortex may participate in post-decision processes rather than model-based decision).

Our computational scheme is quintessentially model-based; however, it permits to dynamically modulate the degree of model-basedness and its temporal horizon. In this scheme, there is no fundamental difference between theta sequences observed during "standard" navigation, and forward sweeps that occur at choice points, and which stretch much farther in space [7]. These are part and parcel of the same model-based (theta-paced) inferential mechanism that runs continuously during navigation (or outside navigation, during sequential processing [81]). Whether or not a more intensive, far-reaching sweep occurs depends on a trade-off between its costs (e.g., costs of computation and the time consumed by it) and benefits (e.g., whether or not engaging in far-looking prediction is useful for action selection). From a computational perspective, one can characterize the benefits of a far-reaching sweep by considering the (epistemic) value of information that it may make accessible [23,52]. This would explain why sweeps predominantly occur at difficult choice points [7], and why repeated exposure to the same contingencies lowers choice uncertainty, thus rendering deep search unnecessary. In keeping, our information-theoretic approach automatically determines the utility of performing a forward sweep and sets its depth, by considering the initial uncertainty and the value of the to-be-acquired information [23,82]. Neurophysiologically, the decision of whether or not to engage in deep search has been hypothesized to involve the prelimbic area (in rodents) [20].

The current MB-RL implementation has a number of limitations, which we briefly summarize here. First, the neurophysiological implementation of internally generated neuronal sequences is more sophisticated than our simplified implementation; for example, there are at least two classes of internally generated sequential activity patterns, termed theta sequences (and sometimes forward sweeps) [7,38,83] and sharp wave ripple sequences (also called replay) [84], which have different neurophysiological signatures and possibly different (albeit coordinated) roles; see [13] for a review. Replay activity in the hippocampus has already inspired various methods for improving learning (e.g., experience replay [30]) or hybridizing model-free and model-based computations (e.g., DYNA [29]); understanding neuronal sequential activity in more detail might permit going beyond these metaphorical mappings and potentially design improved algorithms. A second, related limitation of the current model is that only focuses on forward replays and does not take into account backward replay, which may have a separate computational role. Both forward and backward replays are observed at rest periods (e.g., during sleep), suggesting that they are useful for consolidating the internal model [14]. However, recent evidence suggests that reverse replays are more prominent during the awake state, after (novel) reward observations [85,86]. This makes sense if one considers that backward replays may help learning from recent rewards, possibly using some sort of "eligibility trace" [87] to update the current model or the current policy. More broadly, one may conceptualize replays in terms of *epistemic actions* that aim at gathering (the best) evidence to improve the internal model [52]. In this perspective, it becomes plausible that different kinds of memory contents need to be accessed during sleep, before a choice and after obtaining a reward; see [88] for a recent computational characterization of hippocampal replay in terms of prioritized memory access. Endowing the current model with the ability to "direct" (forward or backward) replays to informative memory content—possibly using a mechanism that marks memories or places with saliency information [89]—is an open research objective. Third, since head direction input is used to classify latent states, the resulting place fields are directional. While place cells often have some directionality in their fields, our model does not account for non-directional or omnidirectional aspects of the fields. This also implies that in order to select an action, the MB-RL agent needs to know its orientation (using e.g., head direction cells). This design choice was made for the sake of simplicity and has no major implications for the phenomena we were interested in; however, extending the model to obtaining omnidirectional place fields (e.g., as shown in Fig 4, in which place fields are averaged across all directions) would help reproducing more accurately neurophysiological data. Furthermore, our model treats the (hippocampal) state space as flat. An interesting alternative possibility that deserves future investigation is that the hippocampal code is hierarchically organized along a septo-temporal axis, with more temporal cells that encode information that are broader in space (e.g., place and grid cells having larger firing fields [90–92]). Hierarchical organization of the state space can be productively investigated in our framework by rendering the Bayesian nonparametric approach hierarchical, or manipulating its concentration (α) parameter. A final limitation of the current model is that it uses simplified grid cells. Several modeling approaches have been proposed that explain grid cells in terms of (for example) attractors [93,94], oscillators [95] or an eigendecomposition of state space [69], which may be exploited to develop more realistic grid cells within our proposed model.

## Methods

### MB-RL agent: Sensors and action primitives

The MB-RL agent is characterized by position and orientation information, provided as real values, relative to a coordinate system with a unit step size. The y-maze is centered on the origin of the agent’s coordinate system and the overall diameter of the maze is about 16 arbitrary units. The agent could move with constant speed using three action primitives: (i) step 1.5 units forward, (ii) turn 90 degrees to the left and make a 1.5-units step, and (iii) turn 90 degrees to the right and make a 1.5-units step. The effective turn and step size were noisy values (Gaussian noise, σ = 0.1). At each discrete time moment, the agent selects one of the three actions and moves; after each action, it obtains sensory information and (sometimes) reward.

The MB-RL agent receives sensory information from two sensors: a head-direction (i.e., orientation) sensor *h* and (a simplified model of) grid cells *G*; the agent has no further position or proximity sensors. In the rat, head-direction cells located in the postsubiculum part of the HC and in the anterior thalamic nuclei discharge as a function of the horizontal orientation of the head, also in darkness, within a preferred sector extending about 90 degrees that persists even for weeks [96] and are hypothesized to drive place fields [97]. In keeping, our orientation sensory unit *h* provides a discrete, 4-level signal that reduces the true orientation to four non-overlapping 90-degree sectors.

In turn, information about the spatial regularities derives by the so-called grid cells located in the medial entorhinal cortex (MEC) as reviewed in [98]. The discharge pattern of each grid cell resembles a hexagonal grid characterized by spatial frequency (spatial range from, e.g., 0.3 to 3 m), phase, and orientation (see Fig 1C). Several models propose detailed explanations of how this pattern could be produced, e.g., oscillatory interference [95] and attractor networks [93]. Here we used a simplified mathematical model that constructs the typical hexagonal pattern of the grid cells by summing three 2D sinusoidal gratings oriented *π*/3 apart [99]: $G(x,y)=\frac{2}{3}(\frac{1}{3}\sum_{i=1}^{3}\cos\left(k_{i}(r-r_{0})\right)+\frac{1}{2})$, where k_i = 1…3_ stand for 2D oriented ramps with spatial phase *r*_0_ = [*x*_0_,*y*_0_]. We equipped our model with 11 grid cells with spatial frequencies ranging from 2 to 7 cycles per maze (step 0.5) and randomly drawn grid orientation and phase (samples in Fig 1C). Each grid cell provides a binary signal sampled on a unit-step grid. The binary signal from all grid cells was combined multiplicatively to provide a signal *G* with about 500 different levels.

Finally, in order to simplify the recognition of target goal sites, we endowed the agent with an implicit target-recognition mechanism, which provides target identity (but not position, which has to be inferred on the basis of the above sensory information). Upon approaching the target within a unit distance, the MB-RL agent receives reward with a given probability, which depends on the experimental phase (see below).

### MB-RL agent: Architecture

The synthetic agent was implemented as a (Bayesian) model-based reinforcement learning (MB-RL) controller, having three key components (Fig 1D). This and the following figures illustrate the model using the formalism of Bayesian networks, which show the model variables (circles) and the conditional distributions (rectangles) linking them.

#### Latent state categorization mechanism

The latent state categorization mechanism learns to categorize the combined head-direction and grid-cell input signal *x* = *G*×*h* into latent states *s* containing implicit spatial information. This component abstracts the acquisition of place cells [5], which form the model's emergent (latent) state space on top of which state-transition and state-value models are learned. The non-parametric categorization of the internal input signal is implemented using a growing probability distribution *P*_*c*_(*s*|*x*) whereby each novel input signal extends the distribution with a new conditional entry. The new entry is initialized according to a Dirichlet process mixture, also known as the Chinese restaurant process (CRP) [100], with a prior that accounts for the popularity of the categories developed thus far. Critically, this categorization is further updated to account for behaviorally relevant transition contingencies (see later).

#### State-transition model

The state-transition model learns the effects of executing a given action primitive *a* in the emergent latent state-space domain: s × a → s′ where s′ is the expected latent state after the transition. This model is intended to abstract the functions of the hippocampus. It is implemented as a conditional multinomial probability distribution P_M_(s′|s,a)~Cat(θ) parametrized by a Dirichlet conjugate prior θ_s,a_(s′)~Dir(α) that keeps a count of the number of time each state *s*′ is reached when the agent started from state *s* and selected action *a*.

#### State-value model

The state-value model learns the value *r* of each latent state *s* given a goal or target *g*. This model is intended to abstract the functions of the ventral striatum. The *state-value model* defining the reinforcement contingencies is implemented as a conditional binomial probability distribution of reinforcement P_v_(r|g,s)~B(φ) (rewarded: *r = 1*, not rewarded: *r = 0*) parameterized by a Beta-conjugate prior φ_g,s_(r)~Beta(β). For each combination of *g* and *s*, the model (through its conjugate prior) accounts for the number of successes (reward) and failures (no reward) during the entire learning experience (see later for details). Note that at difference with most RL models, the value of states depends on the current goal *g*. This is in keeping with evidence that vStr encodes item-specific values, not just scalar values [8].

### MB-RL agent: Control mechanism

We tested the behavior of the simulated agent in conditions that mimic the experimental manipulations of the animal study [19]. During the *cue conditioning* (*Cue*) and *contextual conditioning* (*Context*) phases, action selection (i.e., the Bayesian inference of the next action) was conditioned on a specific cued target *g*; this corresponds to the fact that, in the animal study, the correct goal location was cued with a light. Rather, during *the context conditioning test* (*CX test*) phase, action selection operated in a “free-running mode” in which the target was not externally cued and the animal was able to navigate and search for a reward freely.

To select an action, the control mechanism first reads the combined input signal *x*_*t*_ from the head-direction sensor and the grid cells and uses the categorization distribution to select the corresponding most likely latent category, or state *s*_*t*_ = *argmax*_*s*_ *P*_*c*_(*s*|*x*_*t*_). Then, assuming a Markov Decision Process, inferential action selection maximizes the likelihood to obtain reward for a given target or goal: *a*_*t*_ = *argmax*_*a*_*P*(*r* = 1|*g*,*s*_*t*_,*a*). Assuming further that the value depends only on the landing state following the selected action, we can factorize the value distribution: P(r|g,s,a) = ∑_s′_ P_v_(r|g,s′) P_M_(s′|s,a). Thus, the control mechanism could use the state-transition model *P*_*M*_(*s*′|*s*,*a*) to simulate action execution, and the state-value model *P*_*V*_(*r* = 1|*g*,*s*′) to evaluate the (immediate) expected outcomes under the various possible actions. Note that in practice, evaluating all the latent states would require high computational costs, especially for a large latent state-space–and such exhaustive evaluation would consider subsequent states that are highly unlikely, e.g., two states that lie in different corners of a maze. A more effective approach would consist in using an approximation that only evaluates the state $\tilde{s}$ that most likely would be visited upon executing a given action $a:\;\tilde{s}={argmax}_{s^{\prime }}P_{M}(s\prime |s_{t},a)$; and this approach could generalize to simulated further transitions for deeper value prediction. In the simulations below, we compare the behavior of two control mechanisms: a controller that uses a form of lookahead prediction (forward sweeps) and one that dispenses from using it (baseline).

### Baseline (Shallow) controller

**Fig 8:**
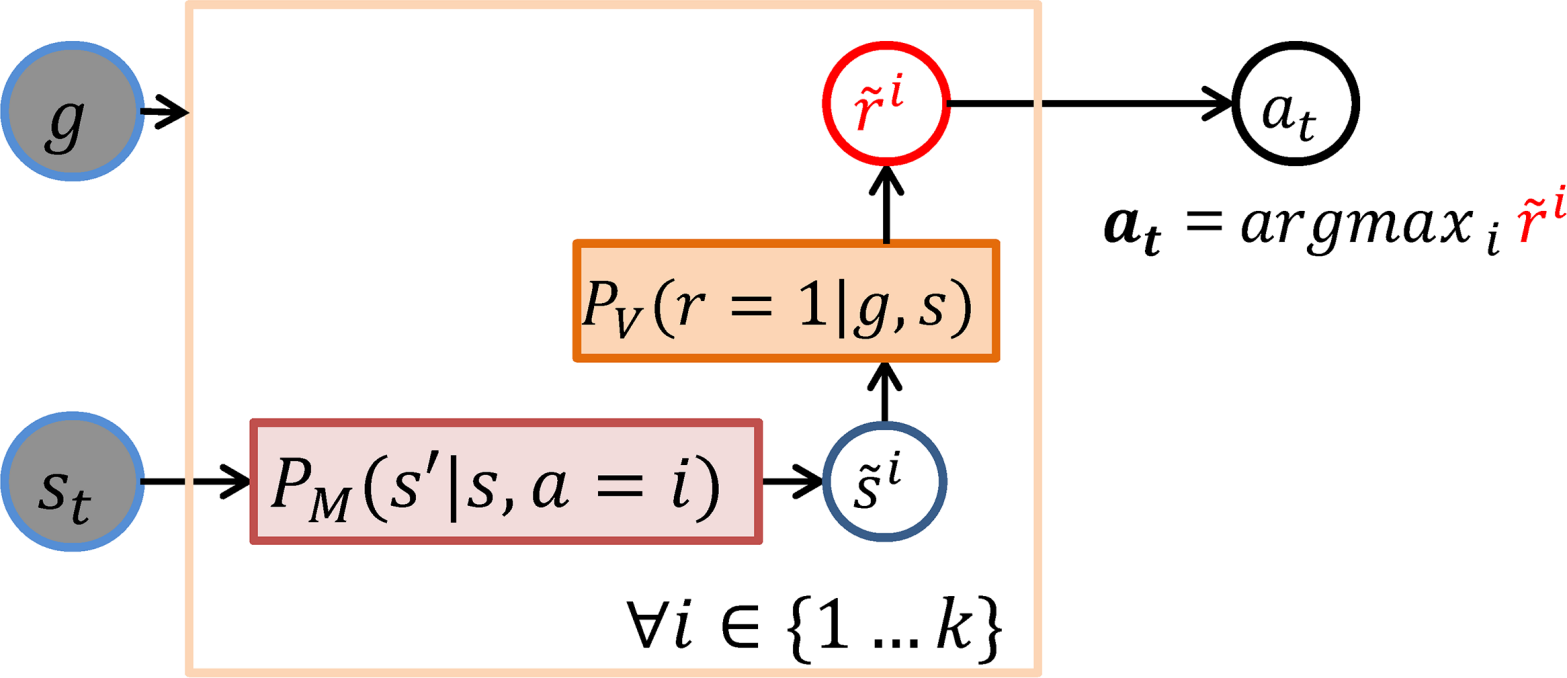
Shallow control mechanism. It exploits the state-transition and state-value models and local maximization to predict the expected value ${\tilde{r}}^{i}$ of each action primitive *a*^*i*^ and select the most valuable one. For each action primitive, the mechanism first finds the latent state that most likely would be achieved by applying that action and then finds among those states the predictably most valuable one. The action bringing to that state is the selected one: *a*_*t*_ = *argmax*_*a*_ *P*_*v*_(*r* = 1|*g*,*argmax*_*s*′_*P*_*M*_(*s*′|*s*_*t*_,*a*)).

The baseline controller shown in Fig 8 selects the action *a*^*i*^ that maximizes the immediately obtainable reward, i.e., $a_{t}=argmax_{a^{i}}P(r=1|g,s_{t},a^{i})$ locally predicted with the help of the state-transition and state-value models: ${\tilde{s}}^{i}={argmax}_{s^{\prime }}P_{M}(s\prime |s_{t},a^{i})$ and $a_{t}={argmax}_{i}P_{V}(r=1|g,{\tilde{s}}^{i})$, which we can combine in a single expression: *a*_*t*_ = *argmax*_*a*_ *P*_*V*_(*r* = 1|*g*,*argmax*_*s*′_*P*_*M*_(*s*′|*s*_*t*_,*a*)). However, this one-step prediction method is quite myopic as it does not consider future events.

### Controller using look-ahead prediction or forward sweeps

**Fig 9:**
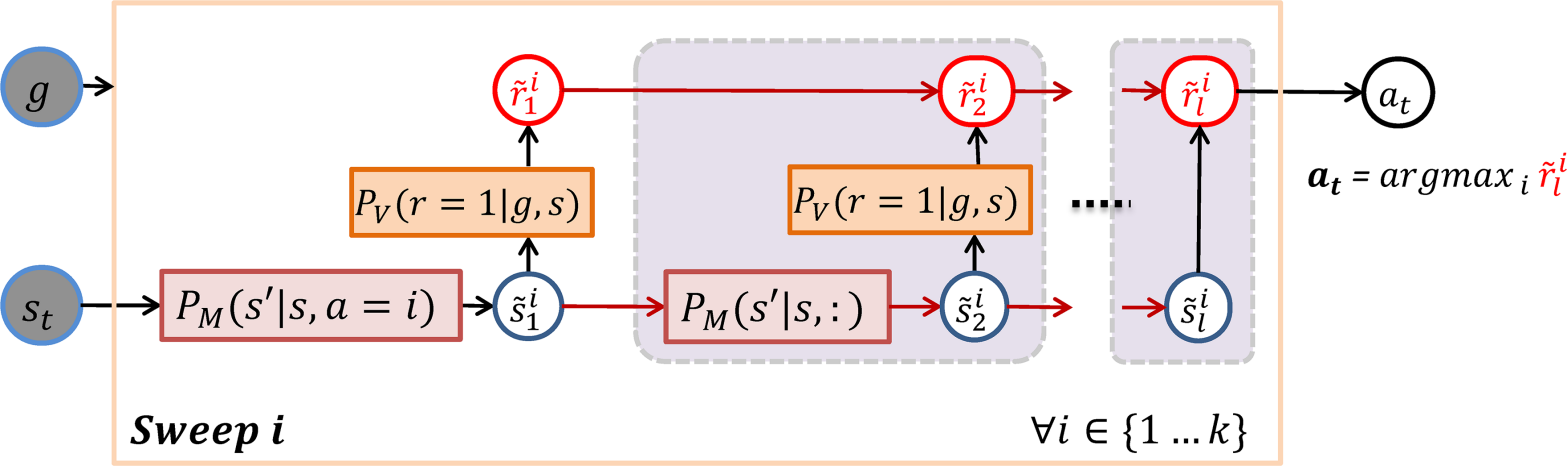
Controller using look-ahead prediction (or forward sweeps). The mechanism includes *k* sweeps, one for each available action primitive, each consisting of *l* steps. At each step *j*, the mechanism first iteratively predicts the next latent state of each sweep $i:\;{\tilde{s}}_{j}^{i}={argmax}_{s\prime }P_{v}(r=1|{g,argmax}_{s\prime }P_{M}(s\prime |{\tilde{s}}_{j-1}^{i},a))$ and then accumulates the predicted value for that state: ${\tilde{r}}_{j}^{i}={\tilde{r}}_{j-1}^{i}+P_{V}(r=1|g,{\tilde{s}}_{j}^{i})$. The first transition of the i-th sweep departs from the current latent state *s*_*t*_ and applies action primitive *a*^*i*^ while the following transitions recursively depart from the predicted state in the previous step and use any action that maximize the predicted reward. Finally, the mechanism selects the action that maximizes the cumulative predicted value: $a_{t}=ar{gmax}_{i}\;{\tilde{r}}^{i}$.

We designed an action selection mechanism based on forward sweeps (Fig 9) that generalizes the idea of local value maximization by using a limited form of forward search: it performs one forward sweep for each available action primitive *a*_*i*_ in order to estimate the future value obtainable as a result of applying this action. For each action *a*^*i*^, the policy first simulates one transition that starts from the current state and applies that action: $s_{t}\times a^{i}\to {\tilde{s}}_{1}^{i}$ where ${{\tilde{s}}_{1}^{i}=argmax}_{s^{\prime }}P_{M}(s^{\prime }|s_{t},a^{i})$. Then it iteratively performs a series of simulated transitions, each one locally maximizing the expected reward without restriction on the action: ${\tilde{s}}_{j}^{i}={argmax}_{s\prime }P_{v}(r=1|g,{argmax}_{s^{\prime }}P_{M}(s\prime |{\tilde{s}}_{j-1}^{i},:))$. Reward evidence ${\tilde{r}}_{}^{i}$ for each action *a*^*i*^ gradually accumulates along each step of each sweep: ${\tilde{r}}_{j}^{i}=\sum_{j=1\ldots l}P_{v}(r=1|g,{\tilde{s}}_{j}^{i})$. The accumulated reward evidence drives stochastic action selection using the *softmax* function (here, with exponent coefficient β = 80).

**Fig 10:**
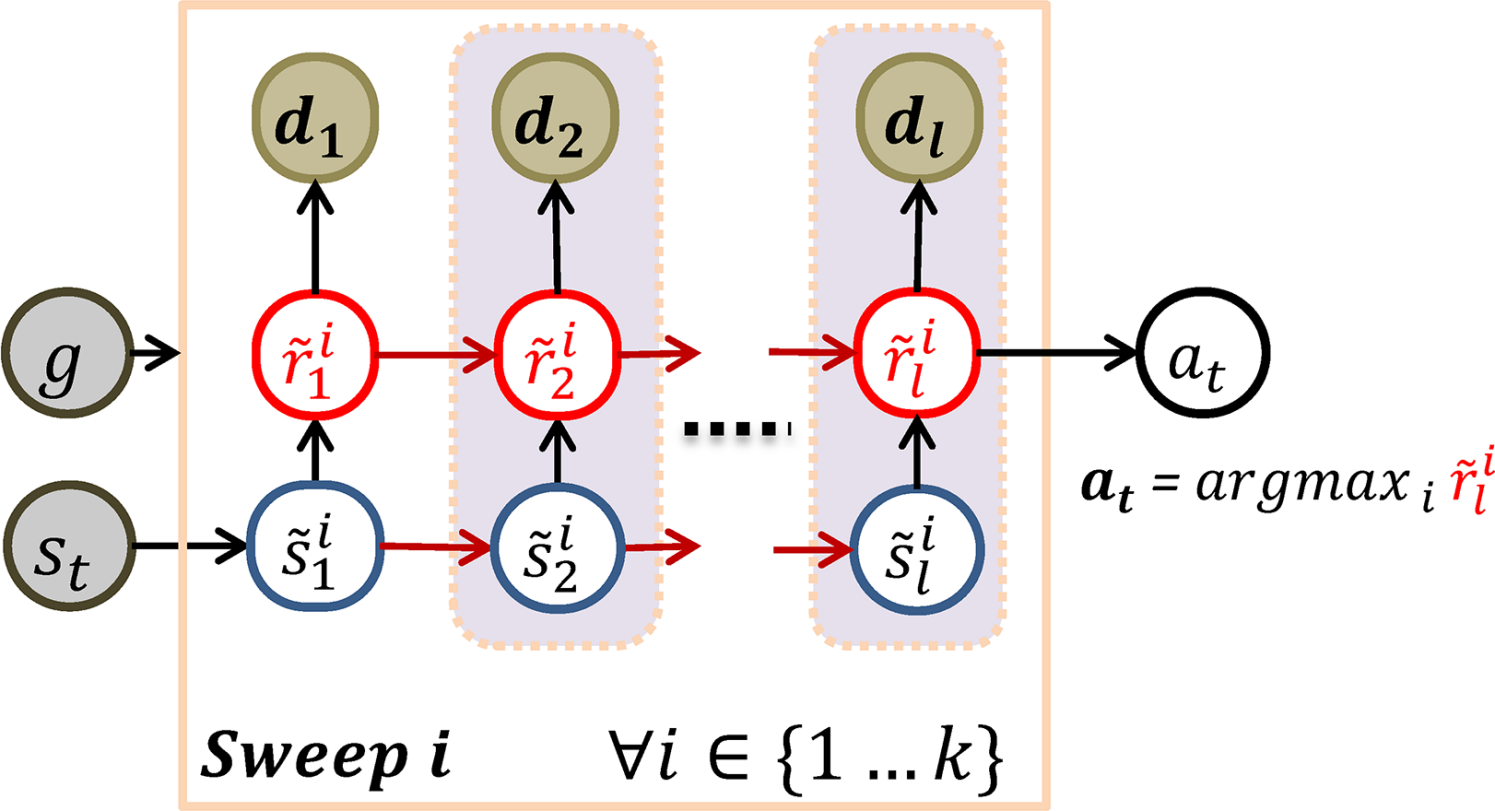
Information-driven adaptive sweep-depth. At each depth *j* is calculated discriminative certainty $d_{j}=\log_{2}\left({\tilde{r_{j}}}^{max1}\right)-\log_{2}\left({\tilde{r_{j}}}^{max2}\right)$ for the two currently most valuable sweeps. Sweep depth increases until the selection certainty exceeds a given threshold: *d*_*j*_ > *d*_*thr*_.

We optimized the efficiency of our sweep-based approach by taking into account a cost-benefit trade-off between the availability of more information and the computational costs required to execute long sweeps. From a theoretical perspective, forward sweeps can be stopped when there is sufficient discriminative evidence for action selection choice [23]. To model this, we defined an information-based measure of *decision certainty* that uses the transition- and value- models to decide which action to simulate and how far to deepen the sweeps (Fig 10). First, the value *v*_*s*_ of being in a state *s* was defined as the negative uncertainty of the probability to obtain reward in that state, i.e., *v*_*s*_ = log_2_ *P*_*v*_(*r* = 1|*g*,*s*). Then, the decision certainty *d*_*ij*_ of taking action *a*^*i*^ (assuming that it brings to state ${\tilde{s}}^{i}$) instead of action *a*^*j*^ (expected that it brings to state ${\tilde{s}}^{j}$) was defined as the difference $d_{ij}=v_{{\tilde{s}}^{i}}-v_{{\tilde{s}}^{j}}$. Thus, to optimize sweep length, at each depth we calculated the decision certainty *d* relative to the two actions with greatest cumulative evidence for reward (see Supporting Information S1 File). Note that the log-difference used to calculate this value implicitly normalizes the accumulated evidence into probabilities. The sweeps were stopped when the decision certainty exceeded a threshold (here, 0.15) and the action with the greatest accumulated evidence for reward was selected.

### MB-RL agent: Learning procedure

**Fig 11:**
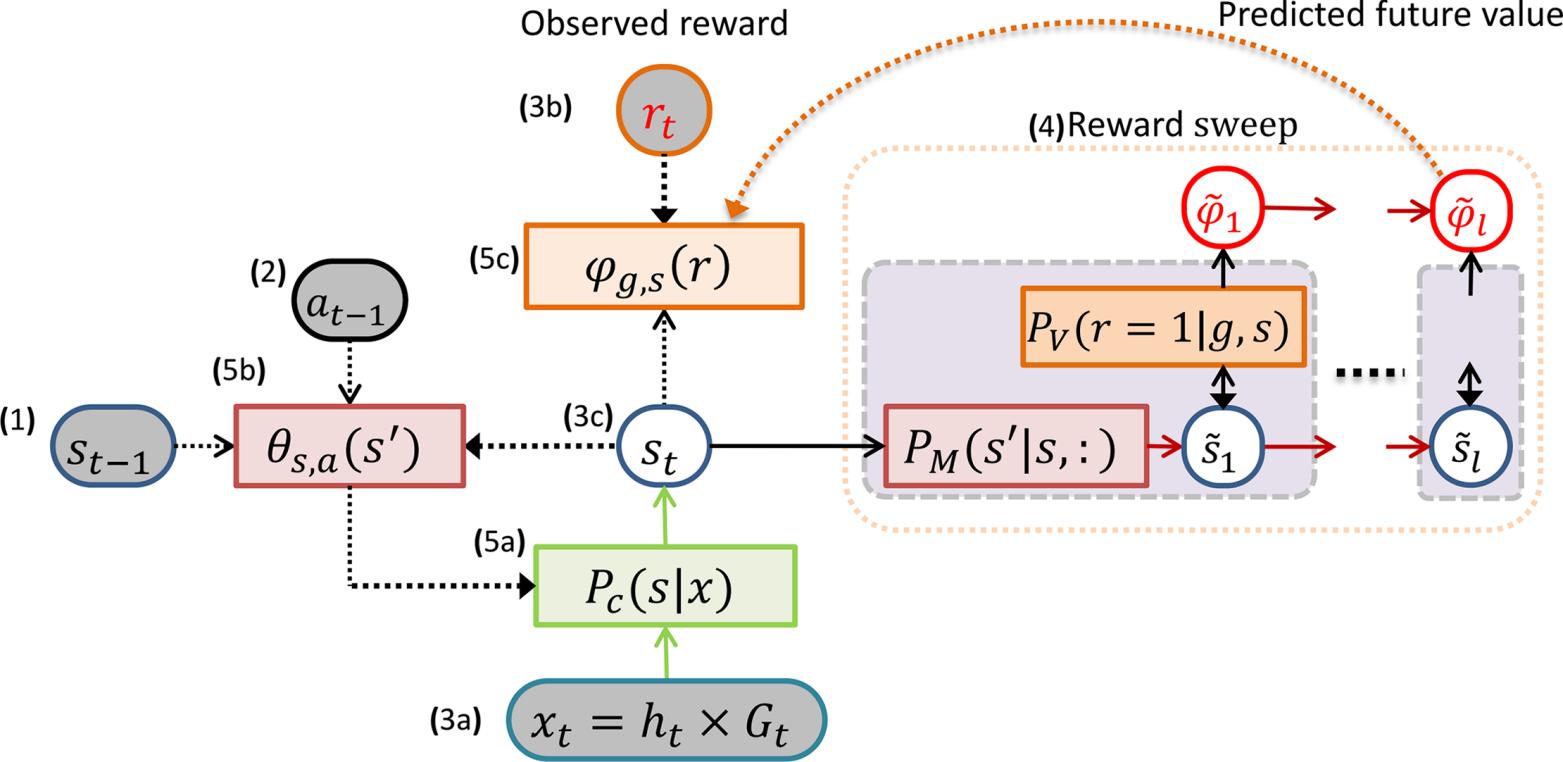
Learning the latent state-space, the state-transition and state-value models. Given (1) the last input *x*_*t*−1_ and latent state *s*_*t*−1_, (2) performed action *a*_*t*−1_, (3) observed new input *x*_*t*_, reward *r*_*t*_, and inferred latent state *s*_*t*_, learning consists of (5) adjusting the categorization model $P_{c}^{t}(s_{i}|x_{t-1})$ to make it more congruent with the state-transition model and updating the conjugate priors $\theta_{s_{t-1},a_{t-1}}(s_{t})$ and $\varphi_{{g,s}_{t}}(:)$ of the state-transition and state-value models to accommodate the internal perception of the experienced behavioral evidence (see the text for details). Notably, the update of the state-value conjugate is a Bayesian analog of TD-learning using predicted discounted future value $\tilde{\varphi }$ accumulated in (4) a forward sweep.

In general, the objective of the MB-RL agent is to obtain maximum reward by moving from a given start-position to a given goal-position, which may vary during the learning process (see later). Following a MB-RL approach, starting from an empty memory, the system has to learn a probabilistic model of the environment (i.e., the transitions from state to state in the maze) and the distribution of reward given the target (or goal), which varies from trial to trial (here, limited to 9 possible goals as in the rodent experiment). Learning thus consists in adjusting the probability distributions of the two (state-state and state-value) agent models on the basis of experience, using Bayes' rule [101]. Importantly, like other deep RL experiments, the model is not provided with a predefined state space, but also has to simultaneously learn to categorize the internal input signal *x* = *G*×*h* into behaviorally useful (spatial) categories *s* that represent the location of the agent in the maze. In sum, the agent has to simultaneously learn three things: state-state and state-value models, and state space; and learning is end-to-end (i.e., all parameters are trained jointly), see Fig 11.

In our simulations, learning consisted of a sequence of trials, each of which started from a random position and had the goal to reach a randomly selected target within 32 time steps. Upon approaching the target within one unit distance and this time limit, the artificial agent received reward with a certain probability. Upon obtaining reward (or after the time limit had expired), a new trial began. Like our animal study [14], learning was divided into two phases that differed on reward distribution. In the first, *Cue Conditioning* phase that lasted for 2.000 trials, reaching a target within the time limit was rewarded with 100%. The starting position in the first 360 trials of this phase was the central area (within 4 units from the center). In the second, *Contextual Conditioning* phase that lasted for 700 trials, reward probability was 75% for the targets in a selected high-reward chamber (fixed for each agent) and 25% for the targets in the other two, low-reward chambers. The amount of reward obtained was increased to maintain the overall reward availability compatible to that in the first phase. The number of simulated learning trials matched those of the animal experiment [19].

In our simulations, we test two different learning procedures: a *baseline* procedure and a *forward sweep* procedure. The two procedures use the same methods for learning the state space and the state-state (transition) models. However, they differ in how they learn the state-value model. They both use the transition model to retrieve the value of possible future states and to update the value function on-line; however, the baseline learning procedure uses a short prediction (equivalent to a sweep of length 1) while the forward sweep procedure uses a longer look-ahead (i.e., sweeps of length 9—a length that is compatible with forward sweeps reported in the literature [7]).

Below we explain in detail how (the probability distributions of) the three components of the MB-RL are learned. All the learning procedures essentially consist in performing Bayesians update of the (conjugate priors of the) probability distributions, in the light of novel observed evidence. Note that hereafter we distinguish the prior from the posterior distributions with the help of additional time-index.

#### State space learning

State space learning uses Bayesian nonparametrics to categorize the input signal, with Chinese Restaurant Process (CRP) priors over the emergent categories [100]. This approach offers an unbounded latent space, but the CRP prior adapts the model complexity to the data by expanding it in a conservative way. Thus, any novel, or unexperienced so far input *x*_*n*+1_ is “assigned” to a new category or state *s*_*new*_ with a small probability P_c_(s_new_|x_n+1_) = α/A (controlled by a concentration parameter α, here α = 20) or to previously initialized latent state *s*_*i*_ according to its popularity across all experienced input states P_c_(s_i_|x_n+1_) = ∑_j_P_c_(s_i_|x_j_)/A where A = α + ∑_i,j_P_c_(s_i_|x_j_) is a normalizing factor. At each time step *t*, an input signal *x*_*t*_ evokes the most probable input-specific latent state $s_{t}=ar{gmax}_{s}P_{c}^{t}(s|x_{t})$ that is in turn used for action selection and learning. Critically, the belief in the category-assignment $P_{c}^{t}(s_{i}|x_{t-1})$ of the last input state *x*_*t*−1_ is scaled for every state *s*_*i*_ with the state-transition contingencies $P_{M}^{t}(s_{t}|s_{i},a_{t-1})$ (adapted from [56,102]):

$$P_{c}^{t+1}(s_{i}|x_{t-1})=\frac{P_{M}^{t}(s_{t}|s_{i},a_{t-1})P_{c}^{t}(s_{i}|x_{t-1})}{\sum_{j}P_{M}^{t}(s_{t}|s_{j},a_{t-1})P_{c}^{t}(s_{j}|x_{t-1})}$$ (1)

This method creates an implicit dependency between state space and state-state (transition) learning, in the sense that states assignment or categorization is retained with higher probability if it is congruent with the emergent transition model. Thus, a novel aspect of our MB-RL approach is that state-transition and state-value learning work in synergy with category- (or state-space-) learning to afford the acquisition of an effective internal model of the experienced interactions with the external environment, from scratch.

Two technical points are worth noticing to contextualize our approach. First, our latent-state model *P*_*c*_(*s*|*x*) is a perceptual model, which permits inferring latent state s (e.g., the current location) corresponding to the current sensory state x. This is distinct from the SR approach [35,69,75,76], which encodes a predictive representation of future states (e.g., of future locations). The two learning methods may seem similar, especially because we use a part of the transition model *P*_*M*_(*s*′|*s*,*a*) to learn a behaviorally relevant space of latent states, see Eq (1). However, it is important to note that we use the probability of the "source" state *s*, not of the "outcome" *s*′, to adjust the perceptual model. This renders the state space and state transition model coherent, but does not yield predictive representations as in the SR approach. In other words, the predictive aspect of the MB-RL agent consists in using model-based, look-ahead prediction, as opposed to using predictive state representations as in the SR approach.

Second, in our simulations, the concentration (α) parameter plays a permissive role in the expansion of the latent state-space: the smaller the α, the smaller the probability that new latent states will be formed. This parameter operates only within the state space learning mechanism and is fully distinct from the parameters of the other model components, such as the (information-driven) mechanism that sets the depth of sweeps explained above, or the discounting factors.

#### State-transition model

After executing an action *a*_*t*−1_, the agent obtains a new observation (sensory measurement *x*_*t*_), infers the corresponding latent state *s*_*t*_, and updates its transition distribution s_t−1_ × a_t−1_ → s_t_, i.e., learns the transition model (see Fig 11). To that aim, the conjugate Dirichlet prior of the conditional multinomial distribution is updated with the new evidence in the latent-space domain: $\theta_{s_{t-1},a_{t-1}}^{t+1}(s_{t})=\theta_{s_{t-1},a_{t-1}}^{t}(s_{t})+1$ and then normalized, to obtain the posterior of the state-transition multinomial $P_{M}^{t+1}(s\prime |s_{t-1},a_{t-1})$.

#### State-value model

The state-value model $P_{v}^{t}(r|g,s)$ is updated using a temporal-difference (TD) like manner that at each learning step accounts for the past experience, or conjugate prior $\varphi_{g,s}^{t}$, observed true reward *r*_*t*_, and predicted future value. The agent makes internal simulations (or sweeps) of length *l* (here, *l* = 1 for the baseline learning procedure and *l* = 9 for the forward sweep learning procedure) conditioned on the latent state *s*_*t*_. During the sweeps, the agent accumulates expected future values in terms of parameters of the conjugate distribution of the model of reward, $\tilde{\varphi }=\sum_{i=1\ldots l}\;{\gamma^{i}\varphi }_{g_{t},{\tilde{s}}_{i}}$, applying a discount factor (here, γ = 0.90). At each step *i* of the sweep, the BM-RL agent exploits a mechanism analogous to the forward sweeps used for control to collect predicted state values. As shown on Fig 11, the mechanism uses the state-transition and the state-value models, and local maximization to infer the latent states of the sweep, i.e., the states with the greatest reward expectancy among all the states that are achievable within one action from the previous state: ${\tilde{s}}_{i}={argmax}_{s\prime }P_{v}(r=1|g,{argmax}_{s^{\prime }}P_{M}(s^{\prime }|{\tilde{s}}_{i-1},:))$ where ${\tilde{s}}_{0}=s_{t}$. Then, the conjugate prior of the conditional binomial distribution of reward is updated in a way that is analogous to temporal difference (TD) learning [1] and which considers the (immediately) observed reward r_t_, and value φ_Obs_ = [1 − r_t_,r_t_] and the (predicted) future value expectancy: $\varphi_{g_{t},s_{t}}^{t+1}=\varphi_{g_{t},s_{t}}^{t}+\alpha (\varphi_{Obs}+\tilde{\varphi }-\varphi_{g_{t},s_{t}}^{t})$. The learning coefficient *α* decreases along with time on a log scale (*α* = *α*_0_/log_10_(*t*), *α*_0_ = 1.5). This learning schedule gradually shifts the value-learning policy, from relying on new evidence to exploiting acquired knowledge. Finally, the posterior is obtained by normalizing the updated conjugate: $P_{v}^{t+1}(:|g_{t},s_{t})=\varphi_{g_{t},s_{t}}^{t+1}(:)/\sum_{r=0..1}\varphi_{g_{t},s_{t}}^{t+1}(r)$.

## Supporting information

S1 File Algorithm of sweep-based action selection. (DOCX)
