# Supplementary material for: Model-based spatial navigation in the hippocampus-ventral striatum circuit: A computational analysis
Source: PLoS Comput Biol. 2018 Sep 17;14(9):e1006316. doi: 10.1371/journal.pcbi.1006316 (PMC6160242; doi:10.1371/journal.pcbi.1006316)
Supplement: S1 File — (DOCX) [file pcbi.1006316.s001.docx]

**Algorithm 1** Sweep-based action selection

**Input**: initial state $s_{t}$, decision threshold $d_{thr}$, transition model $P_{M}\left( s^{'} | s,a \right)$, value model $P_{v}(r|g,s)$

// A. Initialize $n_{a}$ sweeps, one per each action $a^{i}$

1. **for** $i=1:n_{a}$ **do**
2. ${\tilde{s}^{i}=maxarg}_{s^{'}}P_{m}\left( s^{'} | s_{t},a_{i} \right)$ ${\tilde{s}^{i}=argmax}_{s'}P_{M}\left( s' | s_{t},a_{i} \right)$ // Predicted state $\tilde{s}^{i}$ $\tilde{s}^{i}$ upon executing action $a^{i}$
3. ${\tilde{r}^{i}=P}_{v}(r=1|g,\tilde{s}^{i})$ ${\tilde{r}^{i}=P}_{v}(r=1|g,\tilde{s}^{i})$ // Initial evidence for reward $\tilde{r}^{i}$ $\tilde{r}^{i}$upon executing action $a^{i}$
4. **end**
5. $d=\log_{2} \left( \tilde{r}^{max1} \right)-\log_{2} (\tilde{r}^{max2})$ $d=\log_{2} \left( \tilde{r}^{max1} \right)-\log_{2} (\tilde{r}^{max2})$ // decision certainty before starting the sweeps.

// B. Deepen the sweep until insufficient decision certainty

1. **while** $d<d_{thr}$, **do**
2. **for** $i=1:n_{a}$ **do** // For each sweep simulate one transition step
3. $\tilde{s}^{i}=\mathrm{maxarg}_{s'}P_{v}\left( r=1|g,\mathrm{maxarg}_{s^{'}}P_{m}\left( s^{'} | \tilde{s}^{i},: \right) \right)$ $\tilde{s}^{i}=\mathrm{argmax}_{s'}P_{v}\left( r=1|g,\mathrm{argmax}_{s'}P_{M}\left( s' | \tilde{s}^{i},: \right) \right)$ // Most-valuable transition
4. $\tilde{r}^{i}=\tilde{r}^{i}+P_{v}(r=1|g,\tilde{s}^{i})$ $\tilde{r}^{i}=\tilde{r}^{i}+P_{v}(r=1|g,\tilde{s}^{i})$ //Add evidence for the value of action $a^{i}$
5. **end**
6. $d=\log_{2} \left( \tilde{r}^{max1} \right)-\log_{2} (\tilde{r}^{max2})$ $d=\log_{2} \left( \tilde{r}^{max1} \right)-\log_{2} (\tilde{r}^{max2})$ // decision certainty after making a transition
7. **end**

// C. Select the most valuable action.

1. ${a=maxarg}_{i} \tilde{r}^{i}$ ${a=maxarg}_{i}\tilde{r}^{i}$
